# 1245. Knowledge, Attitudes, and Practices of Healthcare Workers towards Antimicrobial Stewardship in Dr. Paulino J. Garcia Memorial Research and Medical Center: A Cross Sectional Study

**DOI:** 10.1093/ofid/ofad500.1085

**Published:** 2023-11-27

**Authors:** Maria Kim Hernandez

**Affiliations:** Dr. Paulino J. Garcia Memorial Research and Medical Center, Quezon City, National Capital Region, Philippines

## Abstract

**Background:**

Antimicrobial resistance, a challenge faced by the public health sector, occurs when pathogens are no longer susceptible to common medicines used to treat them. Several studies have shown that reduction in antibiotic prescription could be done successfully through behavioral intervention. It is important to ensure that antimicrobials are prudently used to preserve its efficacy and enable maximum therapeutic effect. This study assessed the knowledge, attitudes, and practices of healthcare workers towards antimicrobial stewardship at Dr. Paulino J. Garcia Memorial Research and Medical Center in Nueva Ecija, Philippines.

**Methods:**

Cross-sectional design was used in this study. Stratified random sampling and proportional allocation to each stratum was used. Healthcare workers including physicians who routinely prescribed antibiotics; nurses assigned at the wards, and pharmacists were included. Questionnaires were self-administered. Kruskal-Wallis H Test and Dwass-Steel-Critchlow-Fligner pairwise comparisons were used in data analysis.

**Results:**

There were a total of 142 participants. Significant differences were noted on actual knowledge between physicians and nurses, on hand hygiene between physicians and nurses and nurses and pharmacists, on perceived knowledge between surgical area and family medicine and medical area and family medicine. There was also a significant difference between physicians and nurses on attitudes towards antimicrobial stewardship. There were no significant differences noted in the practices of healthcare workers.

**Conclusion:**

Healthcare workers are essential in the fight against antimicrobial resistance. Physicians, nurses, and pharmacists had overall good knowledge, attitudes, and practices towards antimicrobial stewardship but despite this, attention must be given to subsets of the study garnering a poor or moderate level of knowledge. This must be addressed by reinforcing previous known concepts and conducting regular trainings and seminars. Factors affecting the knowledge and practice of antimicrobial stewardship program must also be explored.

**Disclosures:**

**All Authors**: No reported disclosures

